# Superior Tumor Detection for ^68^Ga-FAPI-46 Versus ^18^F-FDG PET/CT and Conventional CT in Patients with Cholangiocarcinoma

**DOI:** 10.2967/jnumed.122.265215

**Published:** 2023-07

**Authors:** Kim M. Pabst, Marija Trajkovic-Arsic, Phyllis F.Y. Cheung, Simone Ballke, Katja Steiger, Timo Bartel, Benedikt M. Schaarschmidt, Aleksandar Milosevic, Robert Seifert, Michael Nader, Lukas Kessler, Jens T. Siveke, Katharina Lueckerath, Stefan Kasper, Ken Herrmann, Nader Hirmas, Hartmut H. Schmidt, Rainer Hamacher, Wolfgang P. Fendler

**Affiliations:** 1 Department of Nuclear Medicine, West German Cancer Center, University Hospital Essen, Essen, Germany;; 2 German Cancer Consortium, partner site University Hospital Essen, Essen, Germany;; 3 Division of Solid Tumor Translational Oncology, German Cancer Research Center, Heidelberg, Germany;; 4 Institute of Pathology, School of Medicine, Technical University of Munich, Munich, Germany;; 5 Department of Diagnostic and Interventional Radiology, University Hospital Essen, University of Duisburg–Essen, Essen Germany;; 6 Department of Nuclear Medicine, University Hospital Münster, Münster, Germany;; 7 Department of Medical Oncology, West German Cancer Center, University Hospital Essen, Essen, Germany;; 8 Bridge Institute of Experimental Tumor Therapy, West German Cancer Center, University Hospital Essen, Essen, Germany;; 9 Medical Clinic B, Department of Gastroenterology, Hepatology, Endocrinology, and Infectiology, University Hospital Münster, Münster, Germany; and; 10 Department of Gastroenterology and Hepatology, University Hospital Duisburg–Essen, Essen, Germany

**Keywords:** ^68^Ga-FAPI-46 PET/CT, ^18^F-FDG PET/CT, conventional CT, cholangiocarcinoma, immunohistochemistry

## Abstract

Management of cholangiocarcinoma is among other factors critically determined by accurate staging. Here, we aimed to assess the accuracy of PET/CT with the novel cancer fibroblast–directed ^68^Ga-fibroblast activation protein (FAP) inhibitor (FAPI)-46 tracer for cholangiocarcinoma staging and management guidance. **Methods:** Patients with cholangiocarcinoma from a prospective observational trial were analyzed. ^68^Ga-FAPI-46 PET/CT detection efficacy was compared with ^18^F-FDG PET/CT and conventional CT. SUV_max_/tumor-to-background ratio (Wilcoxon test) and separately uptake for tumor grade and location (Mann–Whitney *U* test) were compared. Immunohistochemical FAP and glucose transporter 1 (GLUT1) expression of stromal and cancer cells was analyzed. The impact on therapy management was investigated by pre- and post-PET/CT questionnaires sent to the treating physicians. **Results:** In total, 10 patients (6 with intrahepatic cholangiocarcinoma and 4 with extrahepatic cholangiocarcinoma; 6 with grade 2 tumor and 4 with grade 3 tumor) underwent ^68^Ga-FAPI-46 PET/CT and conventional CT; 9 patients underwent additional ^18^F-FDG PET/CT. Immunohistochemical analysis was performed on the entire central tumor plain in 6 patients. Completed questionnaires were returned in 8 cases. Detection rates for ^68^Ga-FAPI-46 PET/CT, ^18^F-FDG PET/CT, and CT were 5, 5, and 5, respectively, for primary tumor; 11, 10, and 3, respectively, for lymph nodes; and 6, 4, and 2, respectively, for distant metastases. ^68^Ga-FAPI-46 versus ^18^F-FDG PET/CT SUV_max_ for primary tumor, lymph nodes, and distant metastases was 14.5 versus 5.2 (*P* = 0.043), 4.7 versus 6.7 (*P* = 0.05), and 9.5 versus 5.3 (*P* = 0.046), respectively, and tumor-to-background ratio (liver) was 12.1 versus 1.9 (*P* = 0.043) for primary tumor. Grade 3 tumors demonstrated a significantly higher ^68^Ga-FAPI-46 uptake than grade 2 tumors (SUV_max_, 12.6 vs. 6.4; *P* = 0.009). Immunohistochemical FAP expression was high on tumor stroma (∼90% of cells positive), whereas GLUT1 expression was high on tumor cells (∼80% of cells positive). Overall, average expression intensity was estimated as grade 3 for FAP and grade 2 for GLUT1. Positive ^68^Ga-FAPI-46 PET findings led to a consequent biopsy workup and diagnosis of cholangiocarcinoma in 1 patient. However, patient treatment was not adjusted on the basis of ^68^Ga-FAPI-46 PET. **Conclusion:** ^68^Ga-FAPI-46 demonstrated superior radiotracer uptake, especially in grade 3 tumors, and lesion detection in patients with cholangiocarcinoma. In line with this result, immunohistochemistry demonstrated high FAP expression on tumor stroma. Accuracy is under investigation in an ongoing investigator-initiated trial.

## Visual Abstract

**Figure:**
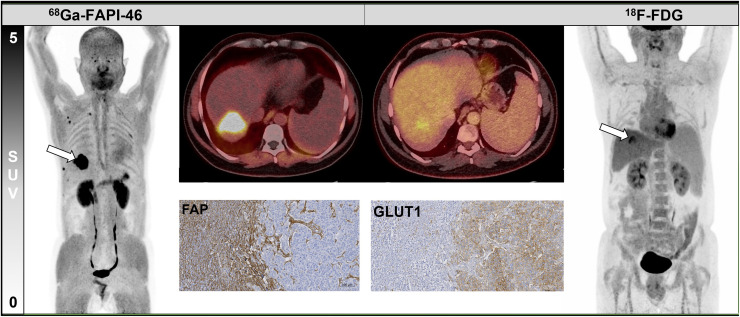

Cholangiocarcinomas originate from intra- and extrahepatic locations of the biliary tract (1). They are the second most common liver malignancy (2), are rising in incidence (3) and are often diagnosed late, frequently leading to a fatal outcome (4). Primary tumors are typically diagnosed by contrast-enhanced and diffusion-weighted MRI with MR cholangiopancreatography (5). Additional imaging by whole-body CT is offered for the detection of distant metastases and vascular involvement (6).

Current guidelines do not routinely recommend PET/CT for the diagnosis and staging of biliary tract malignancies. These recommendations refer to imaging using the radioactive tracer ^18^F-FDG (6). The accuracy of ^18^F-FDG is limited by intertumoral heterogeneous uptake, that is, a high glycolytic rate for high-grade cholangiocarcinoma and a low glycolytic rate for low-grade cholangiocarcinoma (7*,*^8^).

In recent years, quinoline-based fibroblast activation protein (FAP)–specific inhibitors (9) coupled to ^68^Ga have been developed for PET imaging. FAP is expressed by predominantly cancer-associated fibroblasts in the stroma of various tumor entities, leading to highly tumor-specific expression (10).

Because of an abundant tumor stroma whose main cellular components are cancer-associated fibroblasts, cholangiocarcinoma is a promising tumor entity for ^68^Ga-FAP inhibitor (FAPI)-46 PET imaging (11).

Previous publications without a systematic histopathologic workup indicated FAP-directed PET to be highly accurate for the imaging of cholangiocarcinoma (12*,*^13^). Here, we performed a head-to-head comparison of ^68^Ga-FAPI-46 PET, ^18^F-FDG PET, and contrast-enhanced CT and compared the efficacy of these 3 modalities for cholangiocarcinoma detection. Furthermore, we investigated immunohistochemical FAP and glucose transporter 1 (GLUT1) expression from tumor samples of our patient cohort and assessed the impact of ^68^Ga-FAPI-46 PET/CT on cholangiocarcinoma management.

## MATERIALS AND METHODS

### Patient Population

**FIGURE 1.**
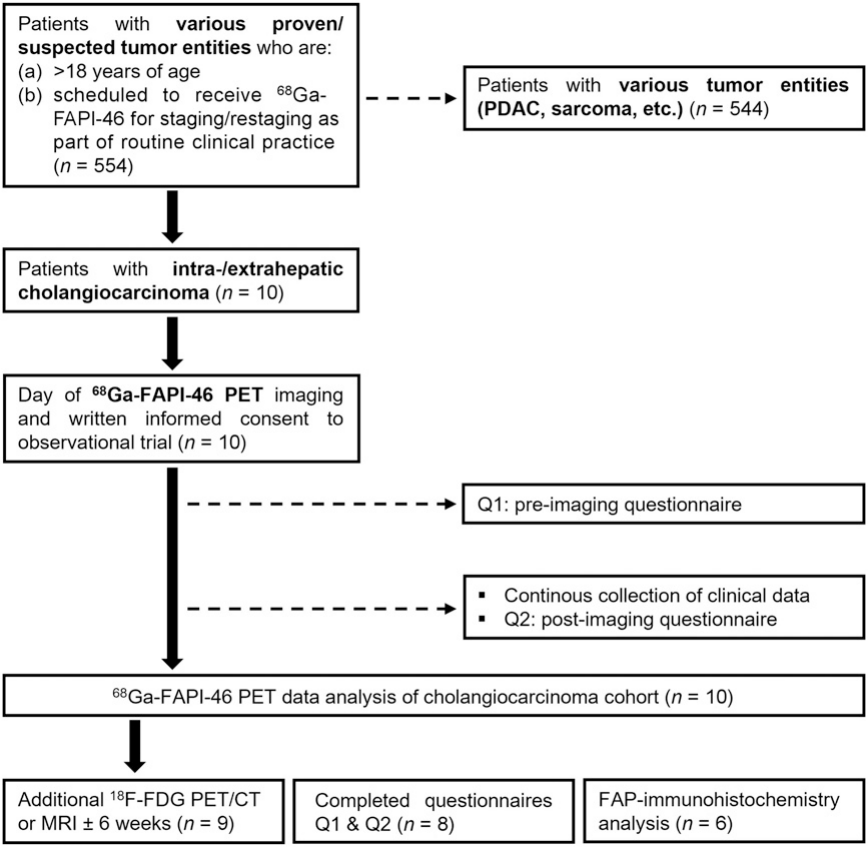
Enrollment flowchart. PDAC = pancreatic ductal adenocarcinoma; Q = questionnaire.

The patient flowchart is shown in Figure 1. This is a subgroup analysis of the ongoing observational trial (NCT04571086) at the University Hospital Essen. Until August 2021, 10 patients with cholangiocarcinoma were included (1.8% of the entire trial). Before enrollment, patients gave written informed consent to undergo ^68^Ga-FAPI-46 PET for a clinical indication. Inclusion criteria were scheduling a ^68^Ga-FAPI PET examination for staging or restaging of cholangiocarcinoma in routine clinical practice and being at least 18 y old. Pregnant, lactating, or breastfeeding women, as well as patients unable to tolerate PET scanning, were excluded. This study was approved by the local Ethics Committee (permits 19-8991-BO and 20-9485-BO).

### Image Acquisition

#### ^68^Ga-FAPI-46 Synthesis and Administration

Radiosynthesis of ^68^Ga-FAPI-46 was described previously (14). Briefly, a pharmaceutical-grade ^68^Ge/^68^Ga generator was applied for the labeling of FAPI-46 using the cassette-based synthesis module Trasis EasyOne.

Patients were not required to be fasting at the time of application and did not require specific preparation. The median intravenously administered activity was 89 MBq (interquartile range [IQR], 79–128 MBq). The median uptake time was 15 min after injection (IQR, 10–38 min). Low-dose CT was performed without application of intravenous contrast medium. Clinical PET/CT scans were obtained in the craniocaudal direction on a Biograph mCT Vision scanner (Siemens Healthineers) (15).

#### ^18^F-FDG PET/CT

^18^F-FDG PET/CT was performed in 8 of 10 (80%) patients and ^18^F-FDG PET/MRI in 1 of 10 (10%). One patient did not undergo additional ^18^F-FDG PET/CT. The median injected activity was 317 MBq (IQR, 266–344 MBq). The median uptake time was 63 min after injection (IQR, 54–80 min after injection). Diagnostic CT was performed, and intravenous contrast medium was given to 6 of 9 (66.7%) patients. The PET protocol was in accordance with the European Association of Nuclear Medicine procedure guidelines for tumor imaging, version 2.0 (16).

#### Conventional CT

Conventional CT was performed on all patients either as part of ^18^F-FDG PET/CT (*n* = 5) or as a stand-alone examination before PET/CT (*n* = 5); the median interval between ^68^Ga-FAPI-46 PET/CT and CT was 17 d (range, 0–36 d). In all patients, diagnostic CT was acquired after application of intravenous contrast medium in the arterial and portal venous phases.

### Image Evaluation

For comparison of ^68^Ga-FAPI-46 and ^18^F-FDG PET/CT, a lesion-based analysis of SUV_max_, SUV_mean_, SUV_peak_, and metabolic tumor volume was performed in consensus by 2 independent, masked readers. For calculation of SUV_mean_ and metabolic tumor volume, volumes of interest were determined by an isocontour threshold of 41% of SUV_max_. Syngo.via software (Siemens Healthineers) was used for measurements of SUV and metabolic tumor volume (16). Lesions visible on only one PET modality were compared with the background of the other PET modality in the same region for statistical reasons. Three regions were selected for evaluation of tumor-to-background ratios (TBRs) using a spheric region of interest: mediastinal blood pool (center of the aortic arch), liver (noninvolved area of the right lobe), and left gluteal muscle (center of the left gluteus). Diagnostic CT was analyzed in consensus by 2 independent, masked radiologists.

### Detection Efficacy

Detection efficacy was assessed through lesion-based evaluation of ^68^Ga-FAPI-46 PET/CT, ^18^F-FDG PET/CT, and conventional CT in 9 of 10 patients. Each detected lesion was considered positive, regardless of the imaging modality. On PET, areas with focal uptake above the background level, not attributable to physiologic findings, were rated positive. On CT, lymph nodes larger than 1 cm in short diameter with suggestive features (contrast enhancement and a round shape, among others) were considered positive. Furthermore, on CT, morphologically delineated or hyperarterialized organ lesions were considered suggestive of malignancy. Follow-up imaging (CT or PET/CT), clinical data, or histologic confirmation were used as the standard of truth.

### Management Questionnaires

To assess changes in intended management after ^68^Ga-FAPI-46 PET/CT, referring physicians completed one questionnaire (questionnaire 1, Supplemental Fig. 1; supplemental materials are available at http://jnm.snmjournals.org) before PET and another questionnaire (questionnaire 2, Supplemental Fig. 2) after reviewing the written ^68^Ga-FAPI-46 PET/CT report.

### Immunohistochemical Analysis of FAP and GLUT1 Expression

Immunohistochemistry was performed on formalin-fixed paraffin-embedded human tissue samples according to the standard laboratory procedures (17). The following antibodies were used: anti-GLUT1 Abcam ab652 (RRID:AB 305540), diluted 1:5,000; anti-FAP α-antibody (SP325); and Abcam ab227703, diluted 1:100. Immunohistochemical expression was evaluated on tumor cells and tumor stroma, and the percentage of intratumoral necrosis related to the tumor areas was also assessed. A simplified visual FAP/GLUT1 grading was applied for stromal and tumor cells, as well as for necrosis. A FAP/GLUT1 grading legend is shown in Table 1. For larger neoplasms, a central slice of the tumor was stained completely. Immunohistochemical analyses were performed on a resection of bioptic samples of the primary or local-recurrence tumors before ^68^Ga-FAPI-46 or ^18^F-FDG PET/CT and consequently do not correspond to visible PET lesions. Two pathologists and 2 biologists performed masked immunohistochemical analysis in consensus.

**TABLE 1.** Visual FAP/GLUT1 Grading

Grade	Definition
0	Absence of FAP/GLUT1 positivity
1	Slight FAP/GLUT1 positivity
2	Moderate FAP/GLUT1 positivity
3	Strong FAP/GLUT1 positivity

### Statistical Analysis

Descriptive statistics and individual patient data are reported. For continuous data, the median, IQR, and range were used. SUV_max_, SUV_mean_, and TBR were compared using the Wilcoxon test. The Mann–Whitney *U* test was performed to compare subgroups for tumor grade and location. To demonstrate the results, visualization with scatterplots was used, with a *P* value of less than 0.05 being considered statistically significant. All analyses were performed using SPSS Statistics (version 27.0; IBM).

## RESULTS

### Patient Characteristics

Overall, 10 patients (6 men and 4 women) were reviewed. The median age was 55.5 y (range, 40–79 y). Included were 6 patients with intrahepatic cholangiocarcinoma and 4 patients with extrahepatic cholangiocarcinoma.

We performed initial staging in 2 patients and restaging in 8. The median interval between diagnosis and initial staging or restaging was 1 mo or 22 mo (range, 5–56 mo), respectively, whereas the median interval was 17 d (range, 0–36 d) between ^68^Ga-FAPI-46 PET/CT and conventional CT and 0 d (range, 0–35 d) between ^68^Ga-FAPI-46 PET/CT and ^18^F-FDG PET/CT. Further clinical information can be found in Table 2.

**TABLE 2.** Patient Characteristics

Patient no.	Age (y)	Sex	Histology	Grade	UICC (initial)	Date of initial diagnosis	Tumor sites on imaging (primary and metastatic)	SUV_max_	
								^68^Ga-FAPI-46	^18^F-FDG
1	58	M	iCC	3	II	01/2020	Bone, lymph node	14.3	6.3
2	79	F	iCC	3	IIIA	01/2021	Liver	17.5	5.1
3	45	M	pCC	2	IIIC	08/2020	Liver	14.5	8.0
4	44	M	iCC	3	II	07/2016	Liver	28.6	5.2
5	57	F	dCC	3	IIIB	03/2018	Ductus hepaticus communis, peritoneal	11.4	11.6
6	70	M	pCC	2	II	12/2019	Liver, lymph node	9.3	4.0
7	40	F	iCC	2	IV	04/2021	Liver, peritoneal, lymph node	25.4	NA
8	79	F	dCC	2	IIB	03/2019	None	NA	NA
9	54	F	iCC	2	IIIA	03/2021	Lymph node	9.8	12.6
10	65	F	iCC	2	IIIB	08/2020	Lymph node	7.7	9.2

UICC = Union for International Cancer Control; iCC = intrahepatic cholangiocarcinoma; pCC = perihilar cholangiocarcinoma; dCC = distal cholangiocarcinoma; NA = not available.

SUV_max_ was determined in hottest lesion for each tracer.

### Detection Efficacy

**FIGURE 2.**
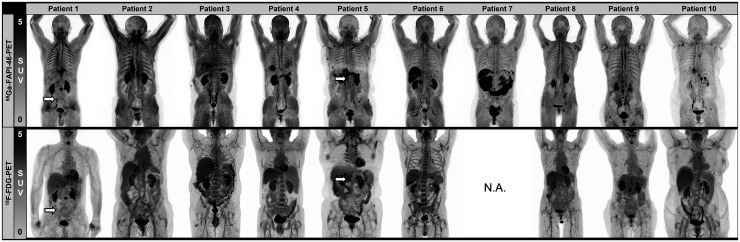
Maximum-intensity projections of ^68^Ga-FAPI-46 and ^18^F-FDG PET for all patients. Tumor lesions that could not be detected by ^18^F-FDG PET are marked with arrows. Tumor sites are listed in Table 2. N.A. = not applicable.

Detection efficacy is summarized in Table 3. Figure 2 shows maximum-intensity projections of all 10 patients. Overall, 22 lesions were detected across all modalities, including primary tumors (*n* =5), lymph node metastases (*n* = 11), and distant metastases (*n* = 6). All primary tumors were detected by all 3 imaging modalities. ^68^Ga-FAPI-46 PET/CT demonstrated the highest detection efficacy for lymph nodes and distant metastases when compared with ^18^F-FDG PET/CT and conventional CT (lymph node metastases: 11 on ^68^Ga-FAPI-46 PET/CT, 10 on ^18^F-FDG PET/CT, and 3 on CT; distant metastases: 6 on ^68^Ga-FAPI-46 PET/CT, 4 on ^18^F-FDG PET/CT, and 2 on CT).

**TABLE 3.** Lesion-Based Detection Efficacy

Location	Overall	Conventional CT	^18^F-FDG PET/CT	^68^Ga-FAPI-46 PET/CT
Primary tumor	5 (100)	5 (100)	5 (100)	5 (100)
Lymph nodes	11 (100)	3 (27.3)	10 (90.9)	11 (100)
Distant metastases	6 (100)	2 (33.3)	4 (66.7)	6 (100)

Data are *n* followed by percentage in parentheses.

### Tumor Uptake

**FIGURE 3.**
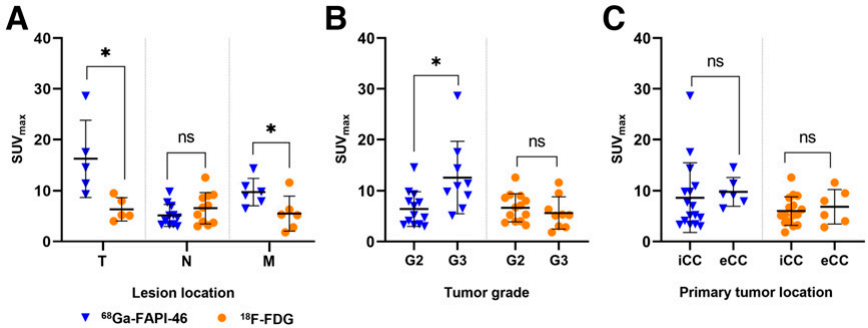
Lesion-based comparison of SUV_max_ between ^68^Ga-FAPI-46 and ^18^F-FDG PET/CT for lesion location (primary tumor, lymph node, distant metastases) (A), tumor grade (B), and location of primary tumor (C). *Statistically significant (*P* < 0.05). eCC = extrahepatic cholangiocarcinoma; G2 = grade 2; G3 = grade 3; iCC = intrahepatic cholangiocarcinoma; M = distant metastases; N = lymph nodes; ns = not statistically significant; T = primary tumor.

Figure 3 summarizes tumor SUV_max_ for ^68^Ga-FAPI-46 versus ^18^F-FDG PET/CT. In total, 22 lesions (6 primary tumors, 11 lymph node metastases, and 6 distant metastases) were assessed. SUV_max_ was significantly higher for ^68^Ga-FAPI-46 PET/CT than for ^18^F-FDG PET/CT for primary lesions (median, 14.5 [IQR, 6.1] vs. 5.2 [IQR, 2.9]; *P* = 0.043) and distant metastases (median, 9.5 [IQR, 2.4] vs. 5.3 [IQR, 2.7]; *P* = 0.046). No significant difference was noted for lymph node metastases (median, 4.7 [IQR, 2.8] vs. 6.7 [IQR, 5.0]; *P* = 0.05). Details are shown in Figure 3A.

Furthermore, tumor uptake for both tracers was investigated with respect to tumor grade (grade 2, *n* = 4; grade 3, *n* = 4) and tumor location (intrahepatic, *n* = 5; extrahepatic, *n* = 3) (Fig. 3B). Two patients were excluded from evaluation because of a missing ^18^F-FDG PET/CT scan or the absence of tumor lesions. ^68^Ga-FAPI-46 SUV_max_ (median, 10.9 [IQR, 5.2] vs. 5.2 [IQR, 4.5]) was significantly higher in patients with grade 3 than grade 2 tumors (Mann–Whitney *U* test, *P* = 0.009). For ^18^F-FDG PET, no significant difference was observed (median, 5.2 [IQR, 3.3] vs. 6.7 [IQR, 4.6]; *P* = 0.33).

SUV_max_ was not significantly different between intra- and extrahepatic cholangiocarcinoma for either ^68^Ga-FAPI-46 (median, 6.1 [IQR, 6.2] vs. 9.2 [IQR, 2.7]; *P* = 0.23) or ^18^F-FDG (median, 5.3 [IQR, 3.6] vs. 6.6 [IQR, 4.8]; *P* = 0.64) (Fig. 3C).

**FIGURE 4.**
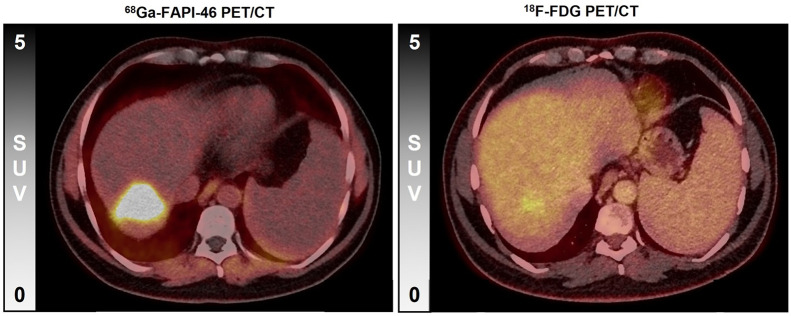
Intrahepatic primary tumor of patient 4, demonstrating 18.7-fold higher tumor-to-background uptake ratio in ^68^Ga-FAPI-46 PET (31.8) than in ^18^F-FDG PET (1.7).

Figure 4 demonstrates a patient example of primary tumor uptake for ^68^Ga-FAPI-46 versus ^18^F-FDG PET/CT, and Supplemental Table 1 shows patient-based, detailed tumor uptake data.

### TBR

**FIGURE 5.**
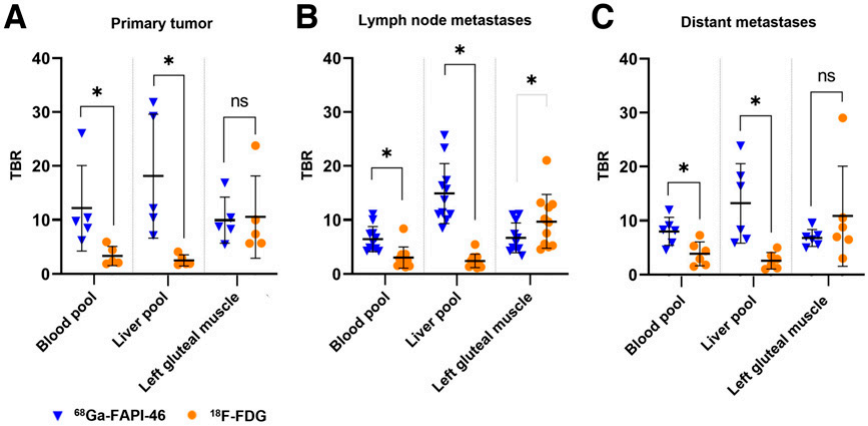
Lesion-based comparison of TBR (blood pool, liver pool, left gluteal muscle; mean ± SD) between ^68^Ga-FAPI-46 and ^18^F-FDG PET for primary tumor (A), lymph node metastases (B), and distant metastases (C). Statistical significance is marked in black for ^68^Ga-FAPI-46 and in gray for ^18^F-FDG. *Statistically significant (*P* < 0.05). ns = not statistically significant.

TBR for mediastinal blood pool, liver, and left gluteal muscle was assessed for both tracers (Fig. 5). For primary tumor, TBR_blood_ (median, 9.7 [IQR, 1.8] for ^68^Ga-FAPI-46 vs. 2.4 [IQR, 2.4] for ^18^F-FDG; *P* = 0.043) and TBR_liver_ (median, 12.1 [IQR, 18.8] vs. 1.9 [IQR, 1.1]; *P* = 0.043) were significantly higher for ^68^Ga-FAPI-46 than for ^18^F-FDG PET, whereas TBR_muscle_ was not significantly different (median, 8.8 [IQR, 2.1] vs. 7.4 [IQR, 4.3]; *P* = 0.69).

Lymph node metastases showed a significantly higher TBR_liver_ (median, 13.7 [IQR, 5.8] vs. 2.3 [IQR, 1.5]; *P* = 0.003) and TBR_blood_ (median, 5.9 [IQR, 2.8] vs. 2.7 [IQR, 1.7]; *P* = 0.004) for ^68^Ga-FAPI-46 PET. In contrast, TBR_muscle_ was significantly higher for ^18^F-FDG PET/CT (median, 5.9 [IQR, 4.0] vs. 9.6 [IQR, 7.1]; *P* = 0.01).

TBR_blood_ (median, 8.2 [IQR, 2.4] vs. 3.7 [IQR, 3.0]; *P* = 0.028) and TBR_liver_ (median, 12.3 [IQR, 10.7] vs. 2.4 [IQR, 2.0]; *P* = 0.028) were significantly higher in ^68^Ga-FAPI-46 PET than ^18^F-FDG PET for distant metastases but not for TBR_muscle_ (median, 6.8 [IQR, 1.3] vs. 7.9 [IQR, 3.5]; *P* = 0.25).

### Change in Management

Eight of 10 questionnaire pairs were completed by the referring physicians. According to the survey, diagnostic tests were not avoided or triggered, and intended therapy did not change in any patient. In 1 patient with an unknown primary, ^68^Ga-FAPI-46 PET/CT localized the tumor. Subsequent biopsy with immunohistochemical analysis led to a cholangiocarcinoma diagnosis.

### FAP and GLUT1 Immunohistochemistry

**FIGURE 6.**
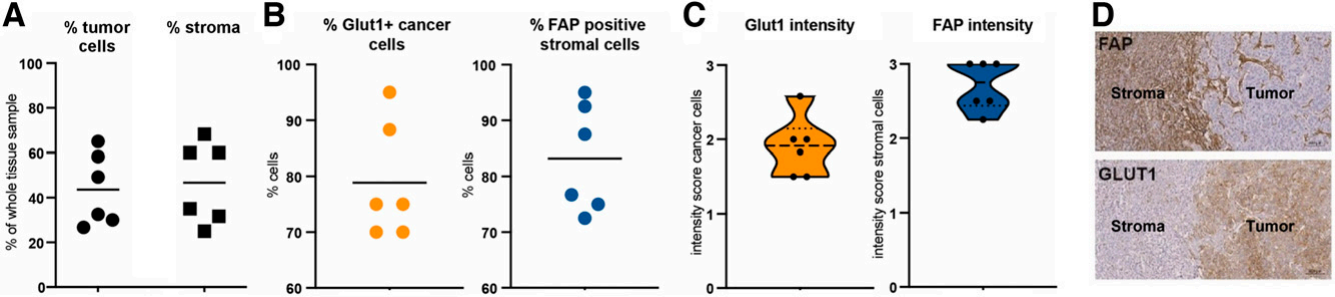
Immunohistochemical FAP/GLUT1 expression graded in accordance with Table 1. (A). Histologic evaluation of tumor cell and stromal content in analyzed samples (2–5 samples per patient, 1 dot presents 1 patient, line presents mean value); tumor cell and stromal content were mostly comparable (∼50%). (B) On average, 90% of stromal cells are positive for FAP whereas 80% of cancer cells are positive for GLUT1. Line presents mean value. (C) Violin plots showing median intensity of 3 for FAP staining on stromal cells but 2 for GLUT1 staining on cancer cells. Line presents mean value. (D) Representative images of immunohistochemistry for FAP and GLUT1 on consecutive sections of 1 patient sample. FAP was strongly expressed in stroma, whereas GLUT1 was detected on tumor cells.

FAP and GLUT1 immunohistochemistry findings are shown in Figures 6A–6C. Surgical samples of primary tumors (*n* = 5) or local recurrences (*n* = 1) from 6 of 10 patients were examined. Figure 6D demonstrates FAP and GLUT1 expression within a tumor sample. According to visual assessment (Table 1), there was a pronounced FAP expression intensity in the tumor stroma (median intensity grade, 3 [range, 2–3]; mean expression of stromal cells, 90% [range, 50%–95%]), whereas there was largely no FAP expression on the tumor cells themselves (median intensity grade, 0 [range, 0–1]; mean tumoral expression, <1% [range, <1%–5%]).

GLUT1 expression was seen predominantly on tumor cells (median intensity grade, 2 [range, 1–3]; mean tumoral expression, 80% [range, 70%–100%]) and only slightly in the tumor stroma (median intensity grade, 0 [range, 0–2]; mean expression of stromal cells, <1% [range, <1%–10%]). Immunohistochemical staining of central tumor slices is shown in Supplemental Figure 3.

## DISCUSSION

Here, we report superior detection efficacy and tumor-to-background uptake for ^68^Ga-FAPI-46 PET/CT versus ^18^F-FDG PET/CT or conventional CT in patients with cholangiocarcinoma. We further demonstrate the impact of ^68^Ga-FAPI-46 PET/CT on diagnostic workup of cholangiocarcinoma in 1 patient.

Currently, the only curative treatment for cholangiocarcinoma is radical surgery of the primary tumor, including lymphadenectomy (6). Patients with unresectable intrahepatic cholangiocarcinoma may benefit from local ablative interventions, such as radioembolization with ^90^Y-microspheres or transarterial chemoembolization (18). In the presence of distant metastases, systemic chemotherapy is the therapy of choice (6). Accurate staging is therefore crucial for management of cholangiocarcinoma.

MRI in combination with MR cholangiopancreatography is the clinical standard for local detection of cholangiocarcinoma (6). According to the guidelines of the European Society for Medical Oncology, additional contrast-enhanced CT determines the relationship between tumor and vasculature (6). Contrast-enhanced CT is currently the imaging modality of choice for staging lymph nodes and distant metastases, although sensitivity and specificity vary significantly across studies (lymph node metastases: sensitivity, 67% [95% CI, 28%–86%]; specificity, 88% [95% CI, 74%–95%]) (19). ^18^F-FDG PET/CT shows advantages in detecting small cholangiocarcinomas as well as lymph node and distant metastases (20–22). However, extrahepatic cholangiocarcinomas and low-grade tumors are difficult to detect because of reduced ^18^F-FDG or a high background signal (8). Here, we show the lowest detection rates for contrast-enhanced CT: we attribute this in particular to the size, exemplified by lymph node metastases, which partly presented at 10 mm or smaller in the investigated cohort.

^68^Ga-FAPI-46 is a novel radioligand that binds to FAP in the tumor stroma and has shown high detection rates for stroma-rich tumors (23). FAP is selectively expressed at high levels by cancer-associated fibroblasts (24*,*^25^) in more than 90% of human epithelial cancers (26).

Recently, Kratochwil et al. reported a high ^68^Ga-FAPI PET SUV_max_ for cholangiocarcinoma (12). In addition, Lan et al. compared detection efficacy for biliary tract cancer of primary tumors, lymph nodes, and distant metastases between ^68^Ga-FAPI and ^18^F-FDG PET/CT and showed ^68^Ga-FAPI to be superior in all 3 subgroups (13). Here, we confirm that ^68^Ga-FAPI-46 PET/CT is superior to ^18^F-FDG PET/CT, and also to conventional CT, for detection of primary tumor but especially for detection efficacy for lymph node and distant metastases.

In addition, ^68^Ga-FAPI-46 PET/CT demonstrates a higher TBR than does ^18^F-FDG PET/CT, which leads to improved delineation, especially of intrahepatic lesions. Notably, ^68^Ga-FAPI-46 PET uptake was highest in grade 3 cholangiocarcinomas, similar to previous findings for ^18^F-FDG PET/CT (8).

Here we, for the first time to our knowledge, present a systematic immunohistochemistry assessment of the imaging cohort. Immunohistochemistry showed high and very specific FAP expression in tumor stroma whereas GLUT1 was expressed mainly on cholangiocarcinoma tumor cells. A high expression level of FAP in tumor stroma was reported previously (23*,*^27^). Cholangiocarcinoma typically presents with a pronounced stromal compartment, which consists mainly of cancer-associated fibroblasts (28*,*^29^). The tumor-specific FAP expression, high stromal content in cholangiocarcinoma and good specificity and retention properties of ^68^Ga-FAPI-46 radioligand probably led to the observed superior TBR and detection rate. In contrast, GLUT1 is a universal glucose transporter that is expressed in many healthy cells in the body, contributing to a higher background level in liver and blood pool that leads to lower TBR ratios and a lower detection specificity for ^18^F-FDG PET.

We could not detect major changes in tumor treatment, mainly because most patients presented for restaging and metastatic stage was already known. With limited therapeutic options for cholangiocarcinoma, the treatment of choice was mostly already performed or planned.

Efficacious treatment options for cholangiocarcinoma are limited (6). In the past decade, target-directed radioligand therapy (RLT) combined with PET, so-called radiotheranostics, has seen unprecedented expansion (30). Theranostic ligands are carrier-bound small molecules that provide diagnostic imaging or therapy depending on the type of radiolabel. Novel RLT has led to prolonged survival in patients with metastatic neuroendocrine tumors (^177^Lu-DOTATOC) (31) and prostate cancer (^177^Lu-PSMA) (32). RLT is characterized by favorable safety and improvement of health-related quality of life (33).

FAP-directed ^90^Y-FAPI and ^177^Lu-FAPI RLT has been reported previously in several tumor entities (e.g., sarcoma, pancreatic adenocarcinoma, and breast cancer) (34–37). ^90^Y-FAPI-46 RLT led to tumor control and was tolerated well in patients with sarcoma or other tumor entities (34*,*^35^). High ^68^Ga-FAPI-46 uptake and strong immunohistochemical FAP expression support the future evaluation of FAP RLT in patients with advanced cholangiocarcinoma.

Our study comes with limitations. ^18^F-FDG PET was mostly combined with contrast-enhanced CT, whereas ^68^Ga-FAPI-46 PET/CT was performed as low-dose CT without a contrast agent. This may affect attenuation correction and SUV quantification. However, Schoen et al. (38) did not find a significant difference with respect to the SUV_max_ of the liver or muscle, for PET/CT with or without contrast enhancement. Other limitations are a small number of patients and the retrospective design. An ongoing prospective interventional investigator-initiated trial (NCT 05160051) aims to assess diagnostic accuracy and target expression in a larger cohort of patients.

## CONCLUSION

In patients with cholangiocarcinoma, ^68^Ga-FAPI-46 demonstrates superior radiotracer uptake, especially in grade 3 tumors, and improved lesion detection when compared with ^18^F-FDG PET/CT. In line with this finding, immunohistochemistry demonstrates high FAP expression in the stroma of cholangiocarcinoma. Superior tumor detection by ^68^Ga-FAPI-46 PET led to tumor diagnosis in 1 patient. FAP targeting may become a valuable option for imaging and potentially RLT of cholangiocarcinoma.

## DISCLOSURE

Kim Pabst has received a Junior Clinician Scientist Stipend from the University Medicine Essen Clinician Scientist Academy (UMEA) sponsored by the faculty of medicine and Deutsche Forschungsgemeinschaft (DFG), travel fees from IPSEN, and research funding from Bayer. Robert Seifert receives research funding from Boehringer Ingelheim Funds and the Else Kröner-Fresenius Stiftung. Timo Bartel receives travel fees from PARI GmbH. Lukas Kessler is a consultant for AAA and BTG and receives fees from Sanofi. Work in the lab of Jens Siveke is supported by the German Cancer Consortium (DKTK). Jens Siveke receives honoraria as a consultant or for continuing medical education presentations from AstraZeneca, Bayer, Immunocore, Novartis, Roche/Genentech, and Servier. His institution receives research funding from Bristol-Myers Squibb, Celgene, Eisbach, Bio, and Roche/Genentech. He holds ownership in and serves on the Board of Directors of Pharma15. Katharina Lueckerath is a consultant for SOFIE Bioscience. Stefan Kasper receives honoraria from Merck Serono, MSD, Novartis, BMS, Amgen, Roche, Sanofi-Aventis, Servier, Incyte, and Lilly and research funding from Merck Serono, Lilly, BMS, and Roche. Ken Herrmann receives personal fees from Bayer, Sofie Biosciences, SIRTEX, Adacap, Curium, Endocyte, IPSEN, Siemens Healthineers, GE Healthcare, Amgen, Novartis, ymabs, Aktis Oncology, and Pharma15; nonfinancial support from ABX; and grants or personal fees from BTG. Rainer Hamacher is supported by the Clinician Scientist Program of the University Medicine Essen Clinician Scientist Academy (UMEA) sponsored by the faculty of medicine and Deutsche Forschungsgemeinschaft (DFG); has received travel grants from Lilly, Novartis, and PharmaMar; and has received fees from Lilly and PharmaMar. Wolfgang Fendler receives research funding from SOFIE Bioscience and Bayer; is a consultant to Janssen, Calyx, and Bayer; is on the speakers bureau for Janssen, Bayer, Novartis, and Telix; and does image review for Parexel. No other potential conflict of interest relevant to this article was reported.

## Notes

KEY POINTS **QUESTION:** Is ^68^Ga-FAPI-46 PET/CT superior to ^18^F-FDG PET/CT and conventional CT in a head-to-head comparison for staging cholangiocarcinoma? **PERTINENT FINDINGS:** ^68^Ga-FAPI-46 PET/CT is superior to both other imaging modalities for detection efficacy, uptake intensity, and TBR. In line with these findings, immunohistochemistry demonstrates high FAP expression of the tumor samples. **IMPLICATIONS FOR PATIENTS CARE:** ^68^Ga-FAPI-46 is a promising novel diagnostic test for staging cholangiocarcinoma. In the future, FAP-directed RLT may represent a new treatment option.
